# Low-cost biosorption of Fe(II) and Fe(III) from single and binary solutions using *Ulva lactuca*-derived cellulose nanocrystals-graphene oxide composite film

**DOI:** 10.1038/s41598-023-33386-7

**Published:** 2023-04-19

**Authors:** Arwa A. Al-Badaani, Awatief F. Hifney, Mahmoud S. Adam, Mohamed Gomaa

**Affiliations:** Botany and Microbiology Department, Faculty of Science, Assiut University, Assiut, 71516 Egypt

**Keywords:** Environmental biotechnology, Nanobiotechnology

## Abstract

The marine algal biomass of *Ulva lactuca* was utilized for the extraction of cellulose and the development of cellulose nanocrystals/graphene oxide film. Cellulose nanocrystals with 50–150 nm were produced by H_2_SO_4_ hydrolysis of the algal cellulose. The adsorption efficiency of the nanocomposite film for Fe(II) and Fe(III) ions was successfully evaluated using Box-Behnken design. The maximum removal for Fe(II) (64.15%) could be attained at pH 5.13, adsorbent dosage 7.93 g L^−1^ and Fe(II) concentration 15.39 mg L^−1^, while the biosorption of Fe(III) was 69.92% at pH 5.0, adsorbent dosage 2 g L^−1^, and Fe(III) concentration 15.0 mg L^−1^. However, in the binary system, the removal efficiency of Fe(II) was enhanced to 95.48% at Fe(II):Fe(III) ratio of 1:1, while the Fe(III) removal was increased to 79.17% at ratio 1:2. The pseudo-second-order kinetics exhibited better fitting to the experimental results of Fe(II) and Fe(III) adsorption in both single and binary systems. The intra-particle diffusion was prominent during the biosorption, but the effect of the external mass transfer was significant. The Langmuir, Freundlich, Langmuir–Freundlich, Temkin, and Dubinin-Radushkevich isotherms showed satisfactory fitting to the experimental data, but they differ in priority based on iron state and pH. The adsorption of Fe(II) in the presence of Fe(III) in a mixture was best represented by the extended Langmuir model, while the extended Langmuir–Freundlich model best fitted the adsorption of Fe(III). The FT-IR analysis indicated that physisorption through electrostatic interaction/complexation is the predominant mechanism for the adsorption of iron using the nanocomposite film.

## Introduction

Water pollution by heavy metals is a serious environmental problem since they are non-biodegradable, persistent and have health, and economic impacts^1^. Iron is one of the most abundant metal elements in the Earth, that is mainly present in variety of rocks, soils and water, both as Fe(II) and Fe(III)^2^. Fe(II) and Fe(III) are important microelements for plants, animals, and microorganisms, but at higher levels they exert various toxic effects. In general, both surface and ground water are vulnerable to iron pollution, but the received concentration varies with the contamination source. Geogenic iron pollution originates from the dissolving of rocks and minerals and leaching into the ground water^3^. Several industrial effluents such as steel tempering, coal, and mining industries are the main sources of iron pollution in the aquatic environment. Landfill leakage, domestic sewage, and wastes from livestock and farms can also contribute to Fe ion water pollution. The two oxidation states of iron can easily change from one to another as a function of pH, temperature, and additional components in the solution.

Iron at concentrations as low as 0.1 mg L^−1^ can cause bad taste in water. Furthermore, iron can increase turbidity of water, stimulate bacterial growth, and can react with other compounds, which catalyze the formation of oxygen radicals and damage cellular DNA^4^. High levels of iron contamination in domestic water can produce stains in laundry, porcelain, etc., along with unpleasant odor^5^. Accordingly, the permissible limit for iron in drinking water as indicated by the World Health Organization (WHO) is 0.3 ppm^3^. Thus, the elimination of iron pollution from aquatic environment is a fundamental process to reduce its adverse effects.

Numerous techniques have been applied effectively to remove heavy metals, such as electrocoagulation, precipitation, reverse osmosis, adsorption and membrane separation^6^. Among these techniques, adsorption is a promising method due to its low operating cost, low secondary pollution and simple equipment requirements^7^.

Cellulose is one of the most abundant natural biopolymers on Earth. Currently, it is utilized for various industries including food packaging, electronics, pharmaceutics, paper, biomedicine, wastewater treatment, cosmetics, textile, construction, food additive, and various aspects of biotechnology^8^. Recently, increased attention has been given to algae-derived cellulose as a promising alternative to plant-derived cellulose^9,10^. In general, the isolation of algal cellulose does not require a delignification step, which reduce the consumed energy and increase cellulose purity compared to that derived from plants^10^. Furthermore, algae are generally characterized by fast growth rates without the need for fertile land and competition with any food resources. Besides, the nanocellulose derived from algae has been reported to have high crystallinity and specific surface area and demonstrate excellent rheological and mechanical properties compared to plant-derived nanocellulose^11,12^.

On the other hand, nanocellulose outstands as one of the most promising, sustainable and eco-friendly nanomaterials for various fields such as biomedical products, environmental remediation, textiles, sensors, catalysis, energy and beyond^8^. Graphene oxide (GO) has been also considered as a promising nanomaterial for application in the environmental technology, energy storage, and medical industries^13^. As an oxidation product of graphene, GO possesses the basic features of graphene, besides various forms of functional groups, which were introduced during the modification process, such as single bond COOH, single bond C, double bond O, and single bond OH. These groups are responsible for its excellent adsorption properties, since they can directly act as anchoring sites for metal ion complexation^14,15^. However, GO cannot be used as adsorbent directly due to its high hydrophilic properties^15,16^. Consequently, a series of graphene oxide composites have been developed to enhance its performance. Furthermore, it has been exploited as a reinforcing agent for cellulose and cellulose nanocrystals for the development of multifunctional nanocomposites. The oxygenated functional groups of GO interact with the OH groups in cellulose via hydrogen bonding leading to the formation of mechanical stable nanocomposite with effective 3D network structure^17^. For instance, Chen^18^ prepared graphene oxide/cellulose hydrogel crosslinked with epichlorohydrin for the adsorption of Cu^2+^, Zn^2+^, Fe^3+^, and Pb^2+^. The use of GO can promote the mechanical strength and thermostability as well as the adsorption properties of the composite hydrogel^17,18^. However, to the best of our knowledge, no attempts have been carried out to utilize algae-derived cellulose nanocrystals and GO for the development of nanocomposite material for the adsorption of heavy metals such as Fe(II) and Fe(III).

The aim of the present study was to develop a low-cost and environment-friendly nanocomposite film based on cellulose nanocrystals extracted from *Ulva lactuca* biomass and graphene oxide for the adsorption of Fe(II) and Fe(III) from aqueous solution. Box-Behnken experimental design was used to improve the efficiency of the adsorption process in terms of different operational parameters (pH, iron concentration, and film dose) in single system. Additionally, adsorption kinetics and isotherms were investigated and compared for the single and binary iron systems.

## Materials and methods

### Extraction of cellulose from *U. lactuca* and preparation of nanocellulose

The isolation of cellulose from *U. lactuca* biomass was performed as described previously^12^. One gram of the extracted cellulose was hydrolyzed using 50 mL of sulfuric acid (64% w/w) at 45 °C and 300 rpm. After 3 h, acid hydrolysis was terminated by adding ten-fold distilled water followed by centrifugation (3800*g*, 10 min) at room temperature. The sample was washed several times until neutrality.

### Preparation of graphene oxide (GO)

Graphene oxide was synthesized from graphite pencil lead^19^. In a typical synthesis, finely powdered graphite pencil lead (2 g) and NaNO_3_ (1 g) were dissolved in 46 mL of concentrated H_2_SO_4_ and stirred in an ice bath for 15 min. Then, 6 g of KMnO_4_ was gradually added into the solution and the temperature was kept below 20 °C. The reaction was stirred continuously in a water bath at 35 °C for 2 h and then maintained at this temperature for 30 min. The prepared solution was slowly diluted by 92 mL of distilled water and the temperature was kept below 98 °C. After 15 min, the mixture was completed to 280 mL using warm distilled water. Then, 20 mL of 30% H_2_O_2_ was added to reduce residual permanganate to soluble manganese ions and stop the gas evolution from the solution. Finally, the suspension was filtered and washed with 5% HCl, followed by washing with distilled water until pH 7. The collected graphene oxide was dried at 60 °C to a constant weight and milled into fine powder using a home blender.

### Preparation of nanocomposite biosorbent

Cellulose nanocrystals (CNC) (0.2% w/v) and graphene oxide (GO) (0.2% w/v) were separately dissolved in distilled water and further homogenized by sonication for 3 min and 5 min, respectively. The film forming solution was prepared by homogenizing 5 mL of GO solution and 5 mL of CNC solution for 1 h at 250 rpm. Then, 5 mL of polyvinyl alcohol (PVA, 5% w/v) was added into the mixture and further homogenized for 30 min. Finally, glycerol was added as a plasticizer (0.3 g g^−1^ CNC) and the mixture was homogenized for 1 h. The film forming mixture (15 mL) was poured into a polyethylene Petri dish and left to dry at 40 °C. The intact film was peeled off and kept in a desiccator at 25 °C for 48 h until further analysis.

### Characterization of CNC/GO film

#### Electron microscopy

The morphology and particle size of CNC and the surface structure of the CNC/GO film were investigated using transmission electron microscope (JEOL JEM-100CX II), and scanning electron microscope (JEOL JSM 5400 LV), respectively at the Electron Microscopy Unit, Assiut University. The particle size of CNC was determined using ImageJ software^20^.

#### Fourier transform-infrared (FT-IR) spectroscopy and determination of crystallinity

FTIR spectra of the developed materials were recorded in the 4000–400 cm^−1^ region using a Nicolet IS 10 FT-IR spectrophotometer. The FT-IR crystallinity was calculated based on the ratio of the absorption bands at 1314–1317 and 1374–1377 cm^−1^^21^.

#### Film thickness and density

The thickness of the nanocomposite film was measured by using a manual micrometer with an error of ± 0.01 mm. The gravimetric weight of film pieces (1 × 1 cm) was measured, and film density was expressed as the ratio of film mass to its volume (the product of area and thickness).

#### Water solubility

The films were dried at 90 °C for 24 h. The pieces of films were immersed in 20 mL distilled water at 25 °C without agitation. After 24 h, the insoluble film fraction was collected by centrifugation (6000 rpm, 5 min) and oven dried (105 °C, 24 h). The water solubility (WS %) was calculated using the following equation:

1 $$\mathrm{Water\,solubility }\left(\mathrm{\%}\right)=\frac{Initial\mathrm{ dry\,weight }-Final\,dry\,weight}{Initial\,dry\,weight}\times 100$$

### Determination of point of zero charge (pH_pzc_)

A series of 10 mL of NaCl solution (0.01 M) with initial pH (pH_i_) set between 2 and 10 were prepared using either HCl or NaOH (1 M). Then, 0.02 g of the developed film was added and allowed to equilibrate for 48 h at room temperature, after that, they were centrifuged (6000 rpm, 10 min), and the final pH (pH_f_) was determined. The pH_pzc_ of the adsorbent was determined from the intersection point of the curve in the plot of pH_f vs_. pH_i_^22^.

### Adsorption studies

Ferrous chloride and ferric chloride stock solutions were prepared in distilled water. Adsorption experiments were performed in 100 mL glass bottles. A known amount of the CNC/GO biosorbent was mixed with a known concentration of Fe(II) or Fe(III). The initial pH of the solution was adjusted to 6 using diluted NaOH and HCl solutions. Biosorption was carried out in an incubator at 25 °C with shaking (100 rpm). An aliquot of 1 mL sample was withdrawn at a predetermined time interval for the determination of residual metal concentration. The concentration of Fe(II) was measured at 520 nm using a spectrophotometer, after the reaction with 100 μL of 1,10 phenanthroline reagent (0.25% w/v)^23^. For the determination of Fe(III), the samples (1 mL) were first mixed with 1 mL of reducing reagent (12.5 mL concentric HCl and 5.5 g L-ascorbic acid, and the total volume was completed to 500 mL using distilled water). After 5 min, 1 mL of chromogenic reagent (68 g Na-acetate and 45 mg 1,10 phenanthroline in 250 mL distilled water) was added and the absorbance was measured at 411 nm^23^. The concentrations of Fe(II) or Fe(III) ions was established using a calibration curve using FeCl_2_ or FeCl_3_, respectively. The percentage removal (*% R*) and the amount of adsorption (*q*_*t*_, mg g^−1^) for Fe(II) and Fe(III) at time t (min) were calculated using the following equation:

2 $$\% R=\frac{\left({C}_{0}-{C}_{t}\right)}{{C}_{0}}\times 100$$

3 $${q}_{t}=\frac{\left({C}_{0}-{C}_{t}\right)\times V}{W}$$

where *C*_0_ is the initial concentration (mg L^−1^) of Fe(II) or Fe(III), *C*_*t*_ is the residual metal concentration at time *t*, *V* represents the volume of the solution (L), and *W* is the dry weight of the nanocomposite film (g).

The adsorption of both Fe(II) and Fe(III) were investigated in single system at different initial concentrations (5, 10 and 15 mg L^−1^), different pH (3—7) and different dosages of CNC/GO film (2, 5 and 8 g L^−1^). In addition, the adsorption of Fe(II) and Fe(III) from a binary mixture was investigated at different ratios: 1:2 (5 mg L^−1^ Fe(II): 10 mg L^−1^ Fe(III)), 1:1 (7.5 mg L^−1^ Fe(II): 7.5 mg L^−1^ Fe(III)), and 2:1 (10 mg L^−1^ Fe(II): 5 mg L^−1^ Fe(III)).

### Experimental design

Box-Behnken experimental design (BBD) was used to investigate and optimize the effect of three factors pH (3–7), initial iron concentration (5–15 mg L^−1^), and the concentration of CNC/GO film (2–8 g L^−1^) on the percentage removal (*% R*) of Fe(II) and Fe(III) from a single component system. The BBD design included 17 experiments with 5 center point replicates to estimate the pure error. The *% R* was expressed as follows:

4 $$\% R=\frac{\left({C}_{0}-{C}_{e}\right)}{{C}_{0}}\times 100$$

where *C*_*0*_ and *C*_*e*_ are the initial and the residual iron concentration at equilibrium, respectively. The BBD was based on a second order polynomial equation of the following general form:

5 $${\text{Y }} = \beta_{o} + \sum \beta_{i} X_{i} + \sum \beta_{ii} X_{i}^{2} + \sum \beta_{ij} X_{i} X_{j}$$

where *Y* is the predicted response; *X*_*i*_ and *X*_*j*_ are the independent adsorption factors; *β*_*o*_ is the regression coefficient of the model; *β*_*i*_, *β*_*ii*_, and *β*_*ij*_ are the linear, quadratic and interaction coefficients, respectively.

### Modeling of adsorption kinetics

Four different kinetic models were employed in the present study to investigate the adsorption of iron ions from single and binary systems viz*.*, pseudo first-order (PFO), pseudo second-order (PSO), Elovich model, and intra-particle diffusion model using the following forms^24–26^:

6 $$\mathrm{Pseudo\,first}{\text{-}}{\rm order\,model}{:}\,{q}_{t}= {q}_{e}(1-exp(-{k}_{1}t))$$

7 $$\mathrm{Pseudo\,second}{\text{-}}\mathrm{order\,model}{:}\,{q}_{t}=\frac{{q}_{e}^{2}{k}_{2}t}{1+{q}_{2}{k}_{2}t}$$

8 $${\mathrm{Elovich\,equation}}{:}\,{q}_{t}=\frac{1}{\beta } \mathrm{ln}(1+\alpha \beta t)$$

9 $${\text{Intra-particle\,diffusion\,equation}}{:}\,{\text{ q}} = k_{i} t^{0.5} + C_{i}$$

where *q*_*t*_ is the amount of iron adsorbed at any time (t) (mg g^−1^), *q*_*e*_ is the amount adsorbed at equilibrium (mg g^−1^), *K*_1_ is the pseudo first-order rate constant (min^−1^), *K*_2_ is the pseudo second-order rate constant (g mg^−1^ min^−1^), *K*_i_ is the intra-particle diffusion rate constant (mg g^−1^ min^−1/2^), *α* is the initial adsorption rate (mg g^−1^ min^−1^), and *β* is the surface coverage (g mg^−1^) during any experiment.

### Modeling of adsorption isotherms

The adsorption mechanisms of Fe(II) and Fe(III) biosorption were analyzed by five different isotherm models including the Langmuir, Freundlich, Temkin and D-R isotherms as described in the following non-linear equations^24,27^

10 $$\mathrm{Langmuir\,isotherm}{:}\,{q}_{e}=\frac{{q}_{m} {K}_{L}{C}_{e}}{1+{K}_{L} {C}_{e}}$$

11 $$\mathrm{Freundlich\,isotherm}{:}\,{q}_{e}={K}_{F}+{C}_{e}^{1/n}$$

12 $$\mathrm{Langmuir}{\text{-}}\mathrm{Freundlich}{:}\,{q}_{e}=\frac{{q}_{m} {K}_{LF}{C}_{e}^{1/n}}{1+{K}_{LF} {C}_{e}^{1/n}}$$

13 $${\mathrm{Temkin\,isotherm}}{:}\,{q}_{e}={B}_{T}\mathrm{ln}({A}_{T}{C}_{e})$$

14 $${\mathrm{where}\,B}_{T}=RT/{b}_{T}$$

15 $$\mathrm{D}{\text{-}}\mathrm{R\,isotherm}\,{q}_{e}={Q}_{s}\mathrm{exp}(-\beta \varepsilon^2)$$

16 $$\mathrm{where }\;\varepsilon =RT\mathrm{ln}(1+\frac{1}{{C}_{e}})$$

where *C*_*e*_ is the equilibrium concentration of iron (mg L^−1^), *q*_*e*_ is the maximum amount of iron adsorbed (mg g^−1^) at equilibrium, and *q*_*m*_ is the maximum adsorption capacity (mg g^−1^). *K*_*L*_ (L mg^−1^), *K*_*F*_ (mg g^−1^ (mg L^−1^)^−1/n^), and *K*_*LF*_ (mg L^−1^)^−1/n^ indicate the Langmuir, Freundlich and Langmuir–Freundlich adsorption constants, respectively. 1*/n* is a heterogenous factor which indicates adsorption intensity or surface heterogeneity. *A*_*T*_ (L mg^−1^) is the equilibrium binding constant relating to the maximum binding energy. *R* is the universal gas constant (8.314 J mol^−1^ K^−1^), *T* is the absolute temperature (298.15 K), and *b*_*T*_ is related to the heat of adsorption (J mol^−1^). Qs is the theoretical saturation capacity (mg g^−1^), and ε (J mol^−1^) is the Polanyi potential.

On the other hand, three extended isotherm models were fitted to the adsorption of Fe(II) and Fe(III) from binary systems as follows^24,27^:

17 $${\text{Extended\,Langmuir}}{:}\,q_{ei} = \frac{{q_{m,i} K_{L,i} C_{e,i} }}{{1 + \mathop \sum \nolimits_{j = 1}^{2} K_{L,j} C_{e,j} }}\,{\text{for}}\quad i = {1},{\text{2 for Fe}}\left( {{\text{II}}} \right){\text{ and Fe}}\left( {{\text{III}}} \right)$$

18 $${\text{Extended\,Freundlich}}\,\,q_{ei} = \frac{{K_{F,i} C_{e,i}^{{\frac{1}{{n_{i} }} + x_{i} }} }}{{C_{e,i}^{{x_{i} }} + y_{i} C_{e,j}^{{z_{i} }} }}\,{\text{for}}\quad i,j = {1},{2}\,{\text{ and}}\,i \ne j$$

19 $${\text{Extended\,Langmuir-Freundlich}}\,q_{e,i} = \frac{{q_{m,i } (K_{LF,i} C_{e,i} )^{{n_{i} }} }}{{1 + \mathop \sum \nolimits_{j = 1}^{2} (K_{{LF,j C_{e,i} )}}^{{n_{i} }} }}\,{\text{for}}\quad i = {1},{2}$$

where *q*_*e*_*,*_*i*_ and *C*_*e*_*,*_*i*_ is the amount adsorbed and residual concentration at equilibrium, respectively for component *i* in the mixture; *q*_*m*_*,*_*i*_ is the maximum adsorption capacity; *K*_*L,i*_, *x*_*i*_, *y*_*i*_, z_i_, and *K*_*LF*_*,*_*i*_ are extended Langmuir, extended Freundlich, and extended Langmuir–Freundlich constants. *K*_*F,i*_ and *n* are Freundlich constants based on single component adsorption.

### Statistical analysis

The experimental design was performed using Design Expert 7.0.0 statistical software (Stat-Ease Inc., USA). Pareto analysis of variance (ANOVA) and response surface plots were generated to visualize the relationships between the independent variables and responses, and the significance was checked by the F- test at probability levels (*p* ≤ 0.05).

The solver function in the Microsoft Excel program was used in the non-linear regression analysis for fitting the experimental data to the different single and binary isotherms. The objective was to minimize the average relative error *(%ARE*, Eq. 18) between the experimental and the predicted results.

20 $$\%ARE= \frac{100}{N} \sum_{i=1}^{N}\left(\frac{\left|{q}_{pred}-{q}_{exp}\right|}{{q}_{pred}}\right)$$

where *q*_*pred*_ and *q*_*exp*_ represents the predicted and the experimental amount of iron adsorbed (*q*), respectively. *N* represents the number of experimental points.

## Results

### Cellulose nanocrystals and film characterization

**Figure 1:**
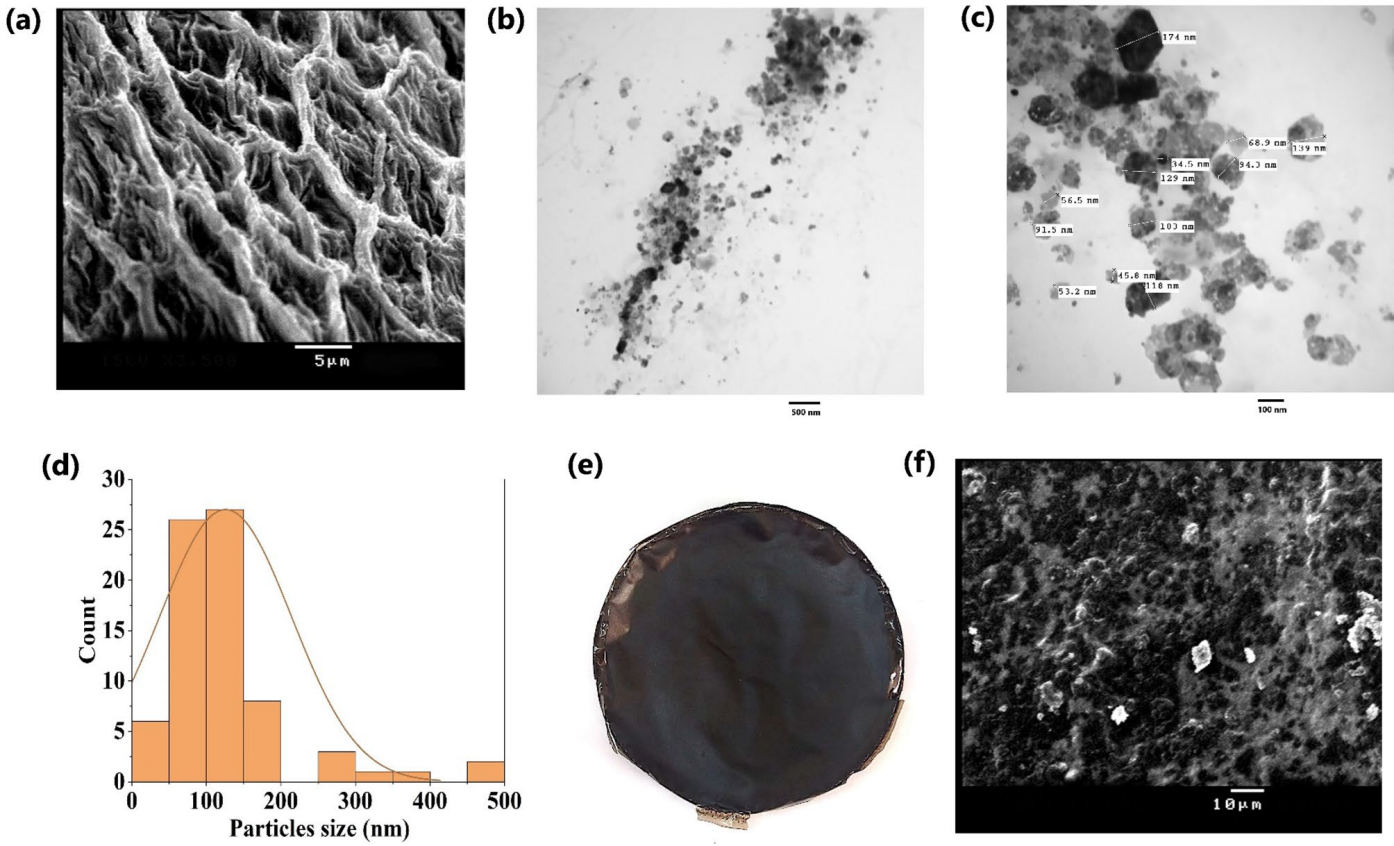
(**a**) SEM image of the cellulose extracted from *Ulva lactuca*, (**b**) and (**c**) TEM images of cellulose nanocrystals, (**d**) Particle size distribution of cellulose nanocrystals, (**e**) Optical micrograph of cellulose nanocrystals-graphene oxide composite film, (**f**) SEM image of the cellulose nanocrystals-graphene oxide film.

The functional characteristics of cellulose and cellulose nanocrystals are generally influenced by their morphological features which are related to the extraction and hydrolysis methods. The pristine cellulose appeared as web-like structures with curled surface (Fig. 1a). The curled surface of the extracted cellulose provides high surface area for the development of composites, and for hydrolysis. Cellulose nanocrystals were developed by removing the amorphous regions and fragmentation of cellulose fibers by H_2_SO_4_ hydrolysis. The hydrolysis and sonication produced spherical to polygonal cellulose nanocrystals (CNC), which are novel and unique (Fig. 1b,c). The particle size of the CNC was predominately in the range 50–150 nm (Fig. 1d). The FT-IR crystallinity degree of cellulose and CNC was 72.58% and 88.73%, respectively.

The developed CNC/GO film was characterized by dark color with homogenous surface (Fig. 1e). The thickness, density and water solubility of the developed nano-adsorbent film were 0.048 ± 0.004 mm, 0.14 ± 0.004 g cm^−3^ and 33.87 ± 1.60%, respectively. The zero point of charge (pH_pzc_) was 7.55 ± 0.07. The film was characterized by a rough surface as indicated by SEM, without the presence of voids (Fig. 1f).

### FT-IR

**Figure 2:**
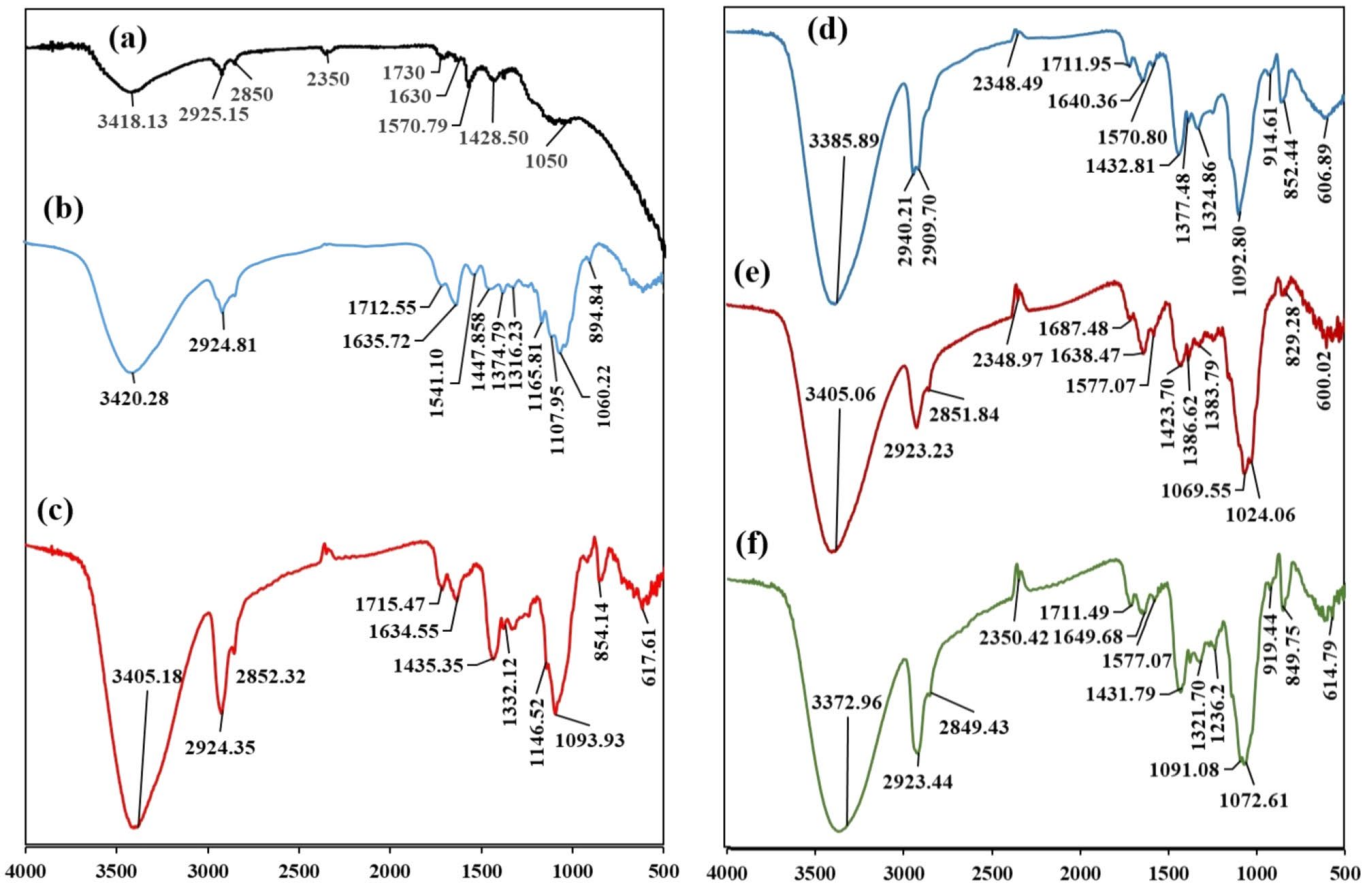
FT-IR spectra of (**a**) Graphene oxide, (**b**) Cellulose nanocrystals, (**c**) Cellulose nanocrystals/graphene oxide film, (**d**) Cellulose nanocrystals/graphene film after Fe(II) adsorption, (**e**) Cellulose nanocrystals/graphene film after Fe(III) adsorption, (**f**) Cellulose nanocrystals/graphene film after binary adsorption of Fe(II) and Fe(III).

The FT-IR analysis was performed to determine the main functional groups in the developed adsorbent and to explain their role in the adsorption process. The characteristic absorption peaks of graphene oxide were observed at 3418.13 cm^−1^ (O–H stretching vibration), 2925.15 and 2850 cm^−1^ (C–H stretching vibrations), 1730 cm^−1^ (carboxyl C=O stretching vibration), 1630 and 1570.79 cm^−1^ (aromatic C=C stretching vibration), and 1428.50 cm^−1^ (carboxy C–O stretching vibration), 1050 cm^−1^ (phenolic C–OH stretching vibration)^1,28^ (Fig. 2a). These signals indicated that the oxygen-based functional groups had been successfully grafted onto the surface and edges of the graphite sheets^29^. On the other hand, the FT-IR spectrum of the algal-derived cellulose nanocrystals was depicted in Fig. 2b. The bands at 3420.28 and 2924.81 cm^−1^ were assigned to –OH groups and CH_2_ stretching vibrations, respectively^30^. The band centered at 1712.55 cm^−1^ was related to the carbonyl (C=O) stretching mode. The band at 1635.72 cm^−1^ was related to the bound H_2_O stretching vibration (Fig. 2b)^12^. Other absorption peak at 1447.85 cm^−1^ was associated with the CH bending of cellulose, and at 1374.79 cm^−1^ was assigned to the C–H deformation^9^. The CH_2_ wagging was detected at 1316.23 cm^−1^ and the polysaccharide absorption band related to the C–O–C symmetrical and asymmetric stretching vibration were detected at 1165.81 and 1107.95 cm^−1^, respectively (Fig. 2b)^9^. The peak at 894.84 cm^−1^ was assigned to the glycosidic ^4^C_1_ ring conformation deformation^31^. The absorption peaks in the developed cellulose nanocrystals at 1447, 1165, and 894 cm^−1^ confirmed the cellulose I structure^31^.

The FT-IR spectrum of the developed CNC/GO film contained similar absorption peaks to pristine cellulose nanocrystals, but with a degree of shifting in their wavenumbers (Fig. 2c). For instance, the band related to the –OH group was shifted to a lower wavenumber in the CNC/GO film, implying a hydrogen bonding interaction between cellulose nanocrystals and graphene oxide (Fig. 2c). Similarly, the C=O, CH_2_, and C–O–C bands were shifted to a higher wavenumber. The peak at 1634.55 cm^−1^ in the CNC/GO film may be assigned to the C=C absorption of graphene oxide^32,33^. The interactions between cellulose nanocrystals and graphene oxide are mainly non-covalent interactions such as hydrogen bonding, electrostatic and Van der Waals forces. Additionally, most of the characteristic absorption peaks of the CNC/GO film were shifted after the adsorption of Fe(II) and Fe(III) in both single and binary mixtures. This observation was mainly related the participation of these groups in the adsorption process (Fig. 2d,e,f).

### Optimization of Fe(II) and Fe(III) adsorption

Three variables (pH, initial iron concentration and adsorbent dosage) were optimized using BBD to investigate the best conditions suitable for the removal of Fe(II) and Fe(III) from their single aqueous solutions (Table 1). Two second order polynomial equations were developed to express the relationship between the % of iron removal and the three studied independent variables as given below.

21 $$\begin{aligned} \% \,{\text{Fe}}\left( {{\text{II}}} \right){\text{ removal }} & = { 22}.{71} + {2}0.{77}A{-}{8}.{63}B + {1}.{28}C{-}0.{579}AB \\ & \quad + {2}.{92}AC{-}{9}.{44}BC{-}{3}.{84}A^{2} + {9}.{32}B^{2} + {14}.{48}C^{2} \\ \end{aligned}$$

22 $$\begin{gathered} \% {\text{ Fe}}\left( {{\text{III}}} \right){\text{ removal}} = {7}.{74} + {11}.{46}A{-}{2}0.{23}B + 0.{9}00C{-}{12}.{1}AB \hfill \\ + {3}.{32}AC + {3}.{11}BC{-}{11}.{24}A^{2} + {15}.{\text{54 B}}^{{2}} + {5}.{66}C^{2} \hfill \\ \end{gathered}$$

where *A*, *B*, and *C* are the independent process variables of pH, iron concentration (mg L^−1^), and adsorbent dose (g L^−1^), respectively.

**Table 1:** Box-Behnken experimental design with coded and un-coded operational variables and the obtained results for the removal of Fe(II) and Fe(III) from aqueous solutions.

Std. order	pH	Iron concentration	Adsorbent concentration	Responses (% removal)	
				Fe(II)	Fe(III)
1	3 (− 1)	5 (− 1)	5 (0)	15.87	30.56
**2**	7 (1)	5 (− 1)	5 (0)	55	65.85
**3**	3 (− 1)	15 (1)	5 (0)	2.5	10.67
**4**	7 (1)	15 (1)	3 (0)	39.36	9.32
**5**	3 (− 1)	10 (0)	5 (− 1)	10.05	13.60
**6**	7 (1)	10 (0)	2 (− 1)	49.32	29.95
**7**	3 (− 1)	10 (0)	2 (1)	11.54	7.73
**8**	7 (1)	10 (0)	8(1)	62.49	37.37
**9**	5 (0)	5 (− 1)	8 (− 1)	48.19	69.06
**10**	5 (0)	15 (1)	2 (− 1)	47.03	26.01
**11**	5 (0)	10 (− 1)	8 (1)	64.82	65.66
**12**	5 (0)	10 (1)	8 (1)	25.95	35.05
**13**^a^	5 (0)	10 (0)	5 (0)	20.39	27.25

^a^Five replicates at the center point.

Based on ANOVA analysis, the developed models were high statistically significant with large F-value (22.71 for Fe(II), and 7.74 for Fe(III) model), and low probability values (*p* < 0.0001 for both models) (Table 2). The coefficient of determination (*R*^2^) exhibited high values (0.983 for Fe(II) and 0.996 for Fe(III)), which implied that most of the observed variability was attributed to the process variables. The adjusted-*R*^2^ values were also high (0.958 for Fe(II) and 0.988 for Fe(III)). Furthermore, the predicted-*R*^2^ values (0.758 for Fe(II) and 0.931 for Fe(III)) were in reasonable agreement with the adjusted-*R*^2^ values, which indicated a good correlation between the observed and predicted values (Table 2).

**Table 2:** Analysis of variance (ANOVA) for response surface quadratic model of cellulose nanocrystals/graphene oxide film.

Source	Fe(II)			Fe(III)		
	CE	F value	*p* Value	CE	F value	*p* Value
Model	22.71	39.11	< 0.0001	27.74	125.96	< 0.0001
A: pH	20.77	213.22	< 0.0001	11.46	181.2	< 0.0001
B: Fe concentration	− 8.63	36.81	0.0009	− 20.23	564.69	< 0.0001
C: Adsorbent concentration	1.28	0.8152	0.4014	0.8996	1.12	0.339
AB	-0.579	0.0829	0.7831	− 12.1	101.03	0.0002
AC	2.92	2.1	0.1971	3.32	7.61	0.0399
BC	-9.44	22.03	0.0033	3.11	6.67	0.0492
A2	-3.84	3.65	0.1047	− 11.24	80.46	0.0003
B2	9.32	21.47	0.0036	15.54	153.77	< 0.0001
C2	14.48	51.79	0.0004	5.66	20.39	0.0063
R2	0.983			0.996		
Adjusted-R^2^	0.958			0.988		
Predicted-R^2^	0.758			0.931		
Adequate Precision	20.906			34.360		
C.V. %	12.31			7.29		

CE: coefficient of estimate.

C.V. %: percentage coefficient of variation.

**Figure 3:**
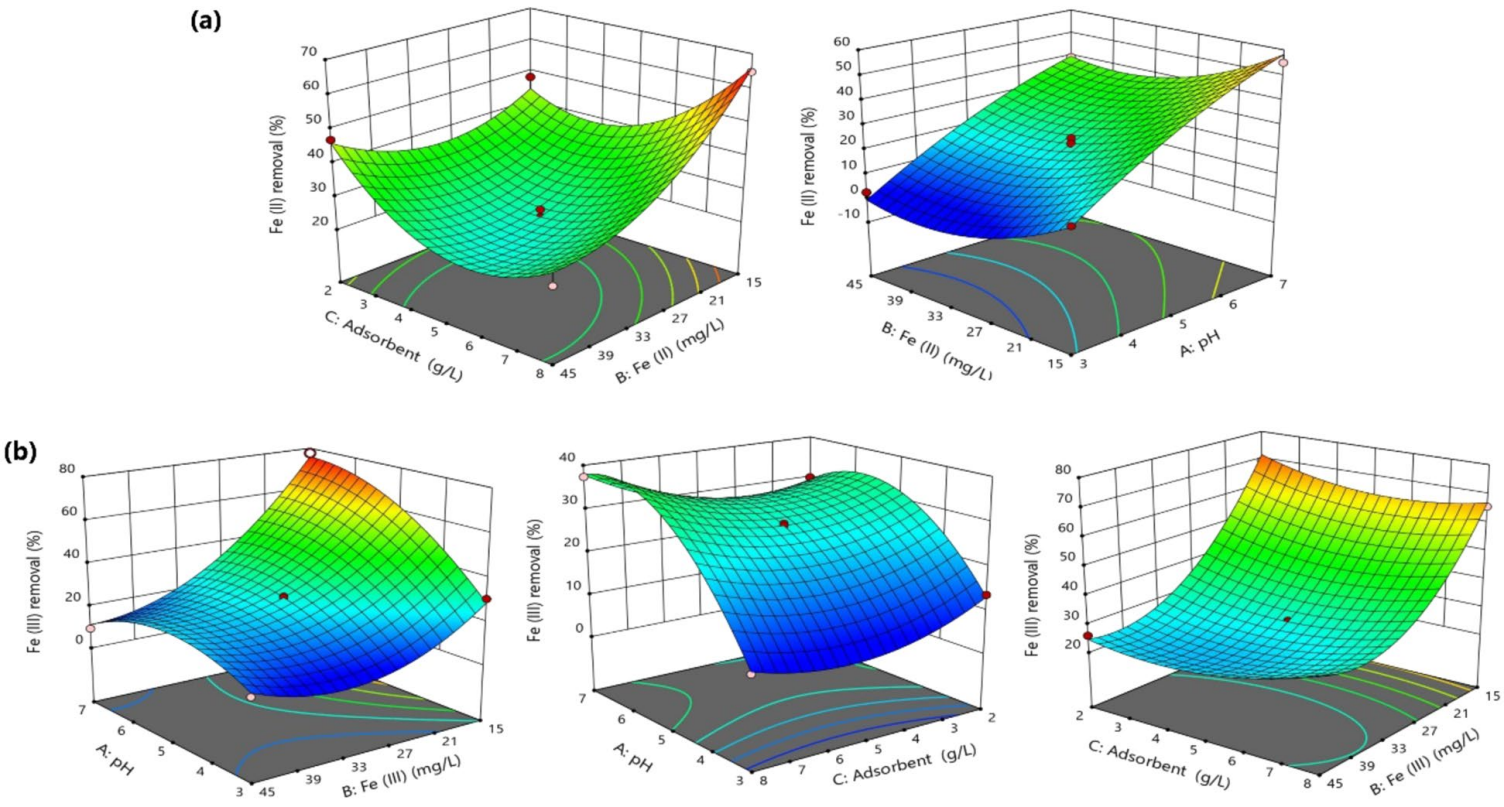
3-D response surface plots for the adsorption of Fe(II) (**a**) and Fe(III) (**b**) using cellulose nanocrystals/graphene oxide film from a single system.

The effects of the independent variables and their mutual interactions were investigated based on the ANOVA analysis and 3-D response surface plots. Initial pH was identified as the most crucial factor affecting on the removal of both iron species from an aqueous solution. The results indicated that the adsorption of iron ions from aqueous solution was significantly dependent on the pH of the solution and the initial iron concentration. In contrast, variations in adsorbent concentration exhibited non-significant effects on the adsorption of Fe(II) and Fe(III) (Table 2). The % removal of Fe(II) and Fe(III) was maximized at high pH values and low initial metal concentrations. Thus, pH exhibited positive effects, while initial metal concentration showed adverse effects. However, pH showed higher effects than initial metal concentration in case of Fe(II), while the opposite trend was observed for Fe(III) (Table 2). Regarding the mutual interactions, adsorbent concentration exhibited significant negative interactive effects with initial Fe(II) concentration, but significant positive interactive effects with initial Fe(III) concentration (Table 2). Therefore, the % Fe(II) removal was increased at low Fe(II) concentration and high adsorbent dose, while the % Fe(III) removal was increased at low Fe(III) concentration and low adsorbent dose (Fig. 3a,b). In addition, pH of Fe(III) solution exhibited significant negative mutual interactions with initial Fe(III) concentration and significant positive interactions with adsorbent dose (Table 2). However, these effects were not evident in case of Fe(II). Furthermore, initial iron and adsorbent concentrations showed positive quadratic effects in case of both iron species, while pH value showed significant negative quadratic effects for Fe(III) only (Table 2).

**Figure 4:**
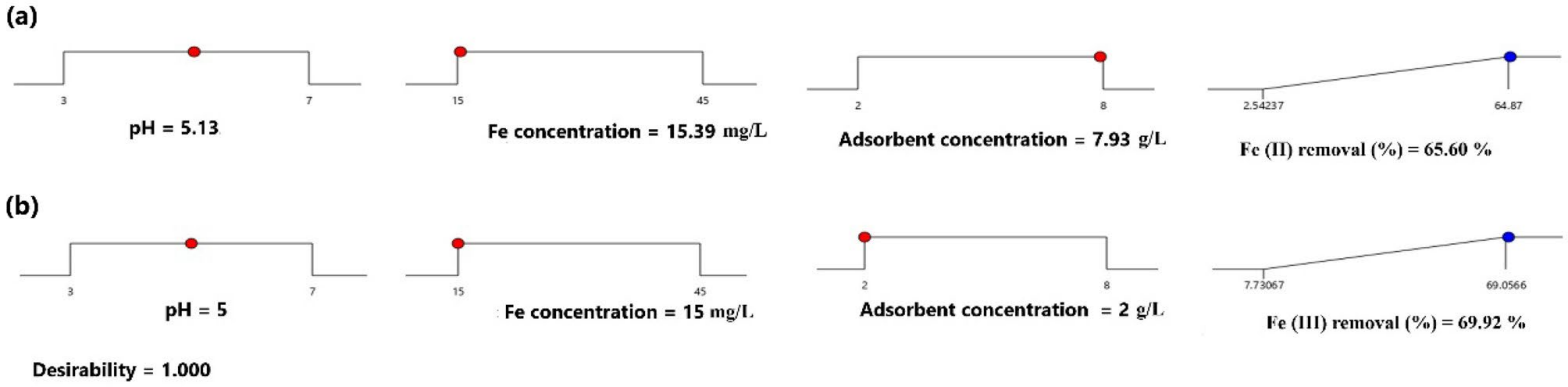
Desirability ramp plot for optimization of (**a**) Fe(II) and (**b**) Fe(III) from single solution.

The optimum conditions to maximize the % removal of Fe(II) and Fe(III) was obtained using Derringer's desirability function in the statistical program (Fig. 4). The optimum conditions for Fe(II) were pH (5.13), initial Fe(II) concentration (15.39 mg L^−1^), and adsorbent dose (7.93 g L^−1^), and the conditions for Fe(III) were pH (5.0), initial Fe(III) concentration (15.0 mg L^−1^), and adsorbent dose (2 g L^−1^). Under these conditions, the predicted % removal values were 65.60% for Fe(II) and 69.92% for Fe(III). Triplicate experiments were performed under the optimum conditions and the experimental values were 64.15 ± 1.10%, 67.92 ± 1.96% for Fe(II) and Fe(III), respectively. Accordingly, the experimental results were in a good agreement with the predicted measurements, which reflected the adequacy and the suitability of the BBD in the optimization of iron removal from aqueous solutions.

### Effect of adsorption parameters

#### Effect of contact time

**Figure 5:**
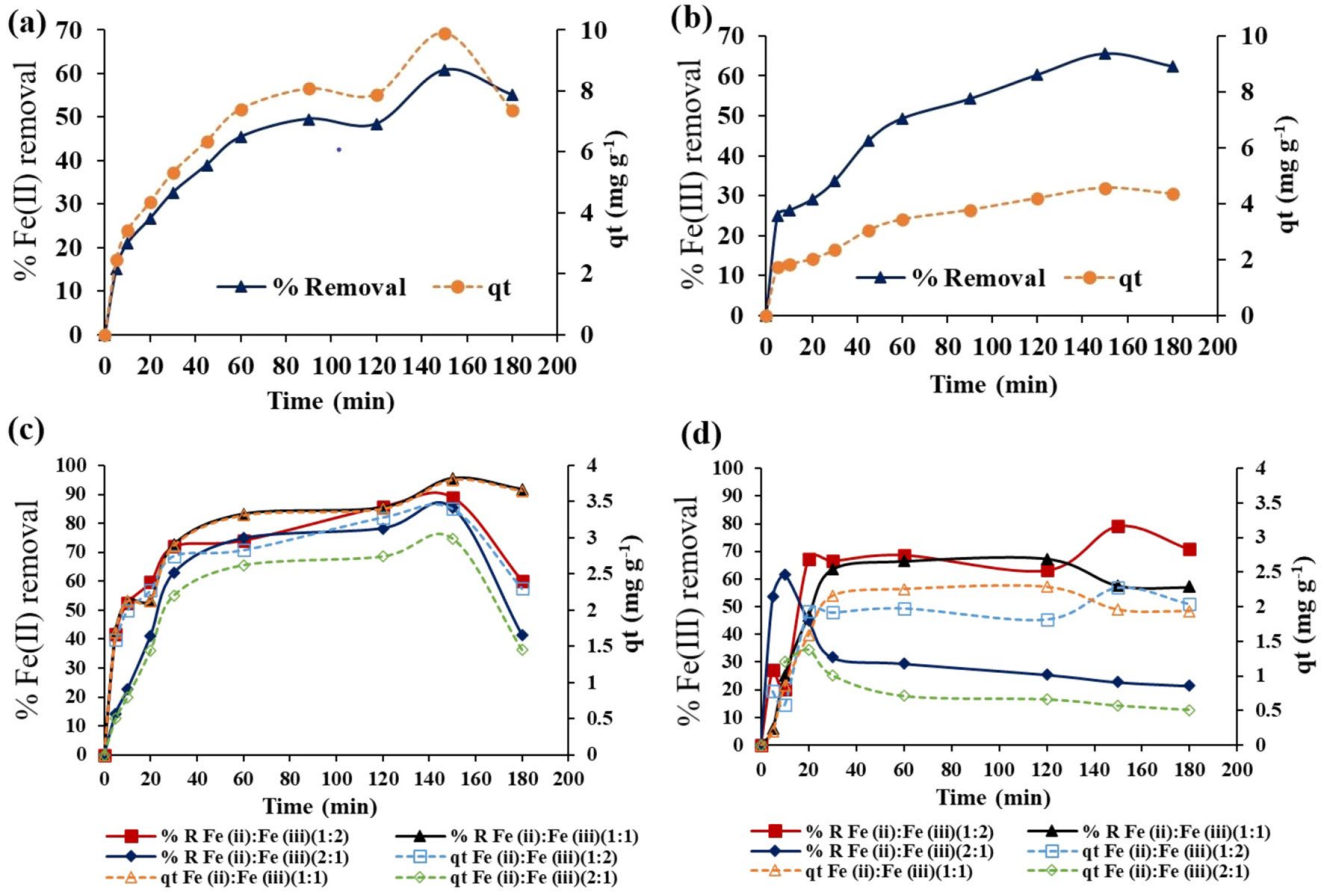
Effect of contact time on the percentage removal (%) and the adsorbed amount (q_t_, mg g^−1^) of Fe(II) and Fe(III) from single (**a**),(**b**) and binary systems (**c**),(**d**) using cellulose nanocrystals/graphene oxide film.

The results of the influence of contact time on the adsorption efficiency (% removal and *q*_*t*_) of Fe(II) and Fe(III) using CNC/GO film were depicted in Fig. 5. The results indicated that the adsorption capacity of Fe(II) and Fe(III) was slow at the initial stages and increased with increasing the contact time and reached maximum value at about 150 min.

On the other hand, the adsorption efficiency of the developed CNC/GO film was investigated in binary systems at different Fe(II):Fe(III) ratios of 1:2, 1:1 and 2:1 (Fig. 5c,d). The effect of contact time on the adsorption of Fe(II) from a binary mixture agreed with the single system, where the maximum adsorption efficiency was observed at around 150 min. However, the adsorption efficiency of Fe(II) in the mixture was ranged from 85.43 at Fe(II):Fe(III) ratio of 1:2 to 95.48% at 1:1 (Fig. 5c), which was relatively higher than the single system (60.78%) (Fig. 5a). Conversely, the maximum adsorption of Fe(III) from a binary mixture was obtained at about 150, 60, and 10 min for Fe(II):Fe(III) ratios of 1:2, 1:1, and 2:1, respectively (Fig. 5d). Furthermore, at Fe(II):Fe(III) ratio of 1:2, the removal efficiency of Fe(III) reached 79.17% (Fig. 5d), which was higher than the single system (65.71%) (Fig. 5b). However, the adsorption of Fe(II) and Fe(III) from single and binary mixtures was characterized by a desorption process, which was observed at longer contact time. At Fe(II):Fe(III) ratio of 2:1, the desorption of Fe(III) began in a short period (> 10 min).

Generally, the adsorption of Fe(III) from a single system was higher than Fe(II), which contradicts the results of the binary mixture.

### Adsorption kinetics

Different kinetic models viz*.* PFO, PSO, Elovich and intra-particle diffusion were fitted to the experimental data and the corresponding coefficient of determination (*R*^2^) and average relative error (*%ARE*) values were used to confirm the satisfactory fitting. The *R*^2^ values of both PFO and PSO models showed a satisfactory fitting to the experimental data for both Fe(II) and Fe(III) biosorption (*R*^2^ > 0.97, Table 3) from single system. However, the PSO model exhibited markedly lower *%ARE* than the PFO model. Similarly, in binary system, the *R*^2^ values for the PSO model were higher than the PFO model in most of the cases (Table 3). In addition, the experimental *q*_*e*_ values for Fe(II) and Fe(III) adsorption were in good agreement with the predicted values by the PSO model in both single and binary mixtures, except in case of Fe(II) in a binary mixture of 2:1 (Fe(II):Fe(III)). Therefore, the PSO equation is better than the PFO model in describing the adsorption of Fe(II) and Fe(III) on the surface of CNC/GO film in both single and binary mixtures. The evaluated *K*_2_ values exhibited similar values in single system (Table 3). While, in binary system, the *K*_2_ values for Fe(III) were combatively higher than Fe(II) at Fe(II):Fe(III) of 1:1 and 2:1, but the opposite trend was observed at 1:2 ratio.

**Table 3:** Kinetic parameters for the adsorption of Fe(II) and Fe(III).

Models	Parameters	Single system		Binary system					
		Fe(II)	Fe(III)	Fe(II)			Fe(III)		
				1:2	1:1	2:1	1:2	1:1	2:1
Experimental values	*q*_*e*_^*exp*^ (mg g^−1^)	9.90	5.56	3.4	3.8	2.99	2.28	1.96	0.48
Pseudo 1st order kinetic	*K*_1_ (min^−1^*)*	0.029	0.015	0.142	0.101	0.034	0.08	0.032	6.77
	*qe*^*cal*^ (mg g^−1^)	9.11	7.61	2.65	3.32	2.94	1.99	2.35	0.52
	*R*^2^	0.971	0.991	0.788	0.740	0.997	0.808	0.842	0.527
	*%ARE*	20.07	31.32	8.96	11.73	5.62	21.52	27.22	26.29
Pseudo 2^nd^ order	*K*_2_ (g mg^−1^ min^−1^)	0.004	0.004	0.043	0.037	0.007	0.038	0.459	0.430
	*qe*^*cal*^ (mg g^−1^)	10.30	6.37	3.32	3.65	4.03	2.43	0.43	0.50
	*h* (mg g^−1^ min^−1^)	0.46	0.15	0.48	0.49	0.12	0.22	0.09	0.11
	*R*^2^	0.988	0.982	0.942	0.876	0.961	0.758	0.933	0.964
	*%ARE*	2.72	22.20	5.08	7.16	6.63	22.20	69.74	34.54
Elovich	*qe*^*exp*^ (mg g^−1^)	9.90	5.56	3.4	3.8	2.99	2. 28	1.96	0.48
	*β* (g mg^−1^)	0.45	1.12	2.04	1.66	1.04	3.40	3.21	4.14
	*α *(mg g^−1^ min^−1^)	0.84	0.83	3.04	2.31	0.17	4.10	5.17	3.16
	*R*^2^	0.970	0.917	0.980	0.942	0.926	0.683	0.760	0.877
	*%ARE*	3.91	12.30	2.19	4.24	11.52	20.14	24.49	50.07
Intraparticle diffusion	*K*_*i*1_ (mg g^−1^ min^−1/2^)	1.36	0.13	0.34	0.32	0.52	0.66	0.60	0.19
	*K*_*i*2_ (mg g^−1^ min^−1/2^)	0.96	0.45	0.10	0.11	0.10	− 0.10	0.02	− 0.30
	*K*_*i*3_ (mg g^−1^ min^−1/2^)	0.15	0.25	− 0.94	− 0.13	− 1.32	0.07	− 0.14	− 0.03
	*R*^2^_1_	0.99	0.99	0.978	0.837	0.973	0.830	0.997	1.00
	*R*^2^_2_	0. 95	0.99	0.958	0.881	0.915	0.705	0.848	0.999
	*R*^2^_3_	0.96	0.99	1.00	1.00	1.00	0.447	0.816	0.984
Distribution coefficient	*k*_*d*_ (L kg^−1^)	251.3	309.4	3072.7	10,971.4	2250.0	2000	4457.14	1530.44

The PSO rate constants were used to calculate the initial adsorption rate (*h,* mg g^−1^ min^−1^) using the following equation:

23 $${h=K}_{2}{q}_{e}^{2}$$

The results indicated that *h* values for Fe(II) was higher than Fe(III) in both single and binary solutions, which indicated an increased recognition selectivity of Fe(II).

In the Elovich model, the constants *α* and *β* are related to initial adsorption rate and the surface coverage of the chemisorption, respectively. The Elovich equation showed a satisfactory fitting to both Fe(II) and Fe(III) (*R*^2^ > 0.91 and *%ARE* < 13, Table 3) in single system. However, in the binary case, the Elovich model showed only satisfactory fitting to the experimental data of Fe(II). The *α* values were generally similar for Fe(II) and Fe(III), while the *β* value was higher for Fe(III) than Fe(II) in single system. In contrast, the *α* and* β* values for Fe(III) were higher than that of Fe(II) in a binary system (Table 3).

**Figure 6:**
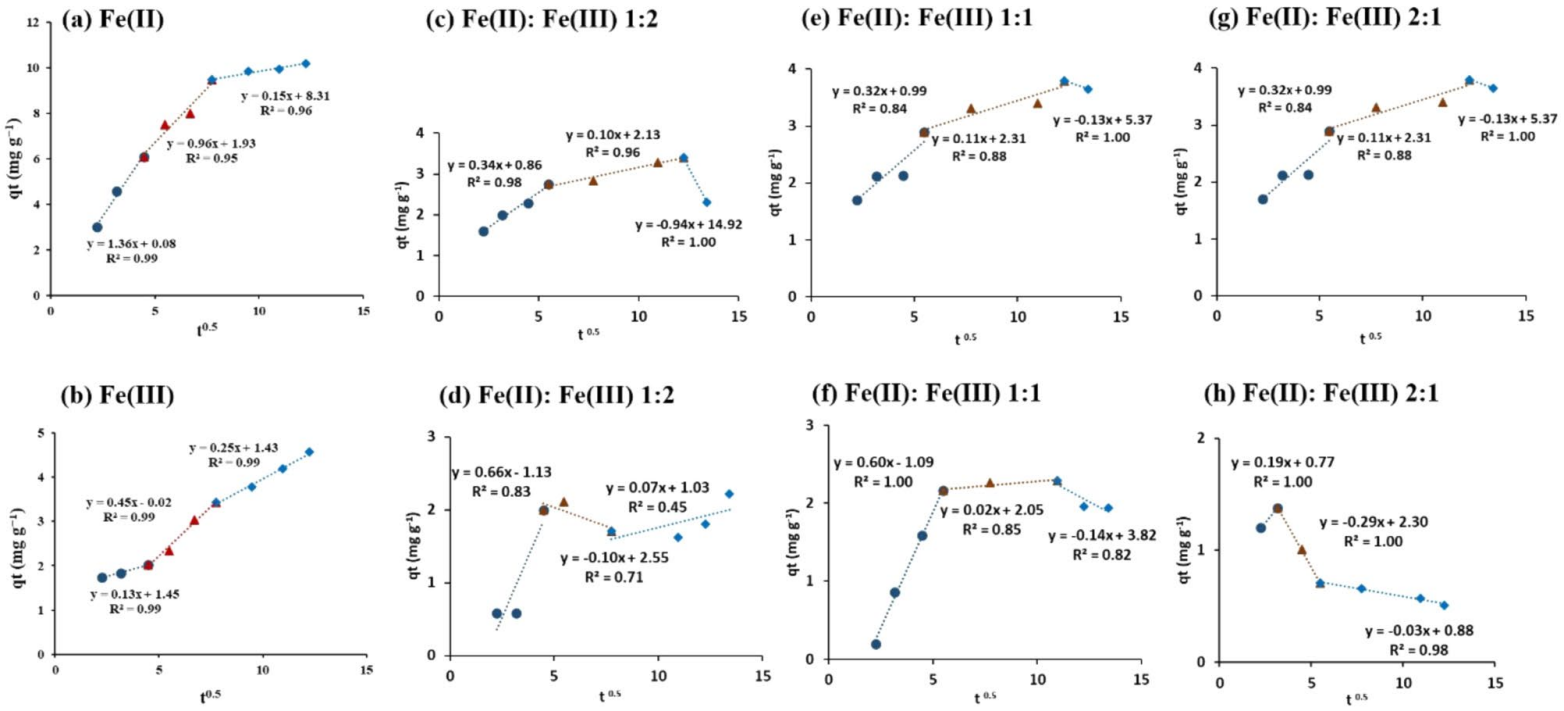
Intra-particle diffusion plot for the adsorption of Fe(II) and Fe(III) by cellulose nanocrystals/graphene oxide film in single and binary systems.

The kinetics of Fe(II) and Fe(III) adsorption were also fitted to the intra-particle diffusion model to determine the rate limiting step, and the results were depicted in Fig. 6. The plots (*qt vs t*^0*.*5^) were characterized by multilinearity with three distinctive linear regions in case of the single system. The three regions were characterized by high *R*^2^ (*R*^2^ > 0.94, Table 3), which implied that the intra-particle diffusion process was prominent during the adsorption of iron ions by the CNC/GO film. The first linear region at the left side indicated the bulk diffusion of the adsorbate molecules into the external surface of the adsorbent. This region exhibited lower rate constants for Fe(III) compared to Fe(II) (*K*_*i*_*,*_1_ = 1.36 for Fe(II), and 0.13 mg g^−1^ min^−1/2^ for Fe(III)) (Fig. 6a,b and Table 3). Furthermore, this step was characterized by short time (up to 20 min). The intra-particle diffusion through the pores of the film was suggested by the second linear region. Similarly, it was observed that the *K*_*i*_*,*_2_ for Fe(II) was higher than Fe(III) (*K*_*i*_*,*_2_ = 0.96 for Fe(II) and 0.45 mg g^−1^ min^−1/2^ for Fe(III)) (Fig. 6a,b, and Table 3). The third linear part was attributed to the equilibrium state, however, it showed higher *K*_*i*_*,*_3_ for Fe(III) than Fe(II) (*K*_*i*_*,*_3_ = 0.15 for Fe(II) and 0.25 mg g^−1^ min^−1/2^ for Fe(III)) (Fig. 6a,b, and Table 3).

A similar multilinear plot with three distinct regions was observed for Fe(II) and Fe(III) in case of their binary mixture (Table 3, Fig. 6c–h). In the first region of bulk diffusion, the rate constant for Fe(II) exhibited higher values (*K*_*i*_*,*_1_ = 0.52 mg g^−1^ min^−1/2^) at Fe(II):Fe(III) ratio of 2:1, which contradicts the result of Fe(III). Furthermore, at Fe(II):Fe(III) ratio of 1:2 and 1:1, the *K*_*i*_*,*_1_ values of Fe(III) were relatively higher than Fe(II). On the other hand, the second linear region of the intra-particle diffusion showed similar *K*_*i*_*,*_2_ values for Fe(II) at different concentrations. The rate constants were characterized by negative values in the third linear region as a result of the desorption process of Fe(II), however, the desorption of Fe(III) was generally observed after the bulk diffusion (Table 3). This result may indicate that the intra-particle diffusion was either fast or not evident for the adsorption of Fe(III) in the presence of Fe(II).

### Modeling of adsorption isotherms

#### Single component system

Adsorption isotherms describe the adsorption process and how the adsorbents interact with the biosorbent material. The adsorption results of iron ions at different initial pH values were fitted using Langmuir, Freundlich, Langmuir–Freundlich, Temkin and D-R isotherms and the results were listed in Table 4. All the models showed satisfactory fitting to the experimental values with *R*^2^ ≥ 0.8, however, some models exhibited lower *%ARE* values than others (Table 4). At pH 3 and 5, the highest *R*^2^ value (0.998 at pH 3 and 0.999 at pH 5) for the modeling of Fe(II) was indicated by the D–R isotherm. However, slight variations in the error values (*%ARE* 40.45–44.15%) were observed between the different models at pH 3, but at pH 5, the lowest *%ARE* values were observed in the case of Langmuir, Freundlich, and Langmuir–Freundlich. Conversely, at pH 7 for Fe(II), the D–R isotherm was characterized by the lowest *%ARE* and *R*^2^ values among different isotherms. On the other hand, Langmuir–Freundlich and Temkin isotherms showed best fitting for the adsorption of Fe(III) at pH 3, while at pH 5, the process could be better described using Freundlich, Langmuir–Freundlich, Temkin and D-R equations owing to low *%ARE* values than the Langmuir isotherm. Furthermore, at pH 7, the comparatively low *%ARE* values for the adsorption of Fe(III) were observed in case of Freundlich, Langmuir–Freundlich, and Temkin models (Table 4). Additionally, by comparing the *%ARE* at different pH, the experimental results showed best fitting at pH 5 and pH 7 for Fe(III) and Fe(II), respectively. Therefore, the maximum adsorption uptake (*q*_*m*_) as indicated by the Langmuir and Langmuir–Freundlich models was increased at pH 5 and pH 7 for Fe(III) and Fe(II), respectively. Furthermore, the *q*_*m*_ were higher for Fe(II) compared to Fe(III).

**Table 4:** Isotherm parameters for Fe(II) and Fe(III) adsorption in a single solution at different initial pH values.

Models	Parameters	Fe(II)			Fe(III)		
		pH 3	pH 5	pH 7	pH 3	pH 5	pH 7
Langmuir	*q*_*m*_ (mg g^−1^)	0.31	1.58	4.00	0.38	3.56	1.74
	*K*_*L*_ (L mg^−1^)	0.075	0.902	0.109	0.039	0.036	0.022
	*R*_*L*_	0.96 – 0.95	0.22 − 0.14	0.97 − 0.95	0.98 – 0.99	0.99 − 0.97	0.99– 0.98
	*θ*	0.038 − 0.048	0.258 − 0.376	0.034 − 0.052	0.016 − 0.015	0.009 − 0.028	0.009 − 0.016
	*R*^2^	0.967	0.971	0.995	0.970	0.878	0.986
	*ARE (%)*	44.15	26.97	1.21	47.40	23.56	52.33
Freundlich	*K*_*F*_ (mg g^−1^ (mg L^−1^)^−1/n^)	0.24	1.20	0.59	0.16	0.72	0.56
	1*/n*	0.003	0.069	0.509	0.057	0.273	0.102
	*R*^2^	0.951	0.932	0.999	0.971	0.867	0.967
	*ARE (%)*	40.45	26.76	4.92	46.90	6.92	43.31
Langmuir–Freundlich	*q*_*m*_ (mg g^−1^)	0.26	1.57	3.10	0.48	2.84	2.12
	*K*_*LF*_ (mg L^−1^)^−1/n^	0.76	0.90	0.16	0.92	0.27	0.14
	*n*	1.48	0.95	0.94	1.26	1.85	2.47
	*R*^2^	0.972	0.973	0.990	0.964	0.850	0.970
	*ARE (%)*	41.45	26.95	3.84	19.89	7.21	46.75
Temkin	*B*_*T*_	0.06	0.78	0.94	0.11	0.40	0.16
	*A*_*T*_ (L mg^−1^)	1.54	0.20	0.80	3.30	3.44	3.91
	*b*_*T*_ (kJ mol^−1^)	43.98	3.17	2.63	23.55	6.27	15.27
	*R*^2^	0.951	0.935	0.997	0.967	0.852	0.959
	*ARE (%)*	43.81	30.46	1.11	20.32	7.22	45.98
Dubinin and Radushkevich	*Q*_*S*_ (mg g^−1^*)*	0.25	2.81	1.95	1.74	1.95	1.99
	*β* × 10^−6^ (mol^2^ J^−2^)	14.54	86.21	0.42	77.66	2.40	0.80
	*E* (kJ mol^−1^)	0.52	0.22	3.09	0.23	1.29	2.24
	*R*^2^	0.998	0.999	0.921	0.970	0.800	0.813
	*ARE (%)*	42.56	39.90	13.30	26.58	8.64	62.50

The separation factor (*R*_*L*_) is an essential parameter of the Langmuir isotherm and can be calculated as:

24 $${R}_{L}= \frac{1}{(1+{K}_{L}{C}_{0})}$$

where *C*_0_ is the initial Fe(II) or Fe(III) concentration (mg L^−1^).

The *R*_*L*_ values describe the nature of the biosorption process as unfavorable (*R*_*L*_ > 1), linear (*R*_*L*_ = 1), favorable (*R*_*L*_ < 1), or irreversible (*R*_*L*_ = 0)^34^. The calculated R_L_ values in the present study falls within the range of 0 < R_L_ < 1 (Table 4), which implied that the biosorption of Fe(II) and Fe(III) using the developed adsorbent was favorable at the different pH values.

Furthermore, the Langmuir results were applied to estimate the surface coverage (*θ*), which is defined as the fraction of the available binding sites occupied by the adsorbate molecules at equilibrium, which can be expressed as follows:

25 $$\theta =\frac{{K}_{L}{C}_{0}}{1+({K}_{L}{C}_{0})}$$

The highest *θ* values were observed for the adsorption of bivalent iron compared to its trivalent state. Furthermore, the *θ* values were markedly increased at pH 5 for Fe (II), but in case of Fe(III), a slight variation was observed.

On the other hand, the 1*/n* parameter of the Freundlich equation is applied to estimate the surface heterogeneity or the adsorption strength as linear (1*/n* = 1), physical (1*/n* < 1) or chemical (1*/n* > 1)^25^. The estimated 1*/n* values in the present study were < 1, which implied that the adsorption of iron species using CNC/GO adsorbent was more inclined towards physisorption, and reflected the importance of surface heterogeneity^24^.

The mean free energy of biosorption, *E* (kJ mol^−1^), which defined as the energy transported from the adsorbate to the adsorbent surface, was calculated as:

26 $$E=({2\beta )}^\frac{1}{2}$$

The *E* value provides information on the mechanism of adsorption, where *E* values > 16 kJ mol^−1^ indicate chemisorption, and *E* < 8 kJ mol^−1^ is considered physisorption^35,36^. In the present study, the *E* values were less than 8 kJ mol^−1^ (Table 4), which indicated that the mechanism of the iron adsorption using CNC/GO film was predominately physisorption.

#### Binary component system

Different isotherm models including the extended Langmuir, extended Freundlich, and extended Langmuir–Freundlich were fitted to the experimental data for the adsorption of iron species from binary systems (Table 5). In case of Fe(II), the lowest *%ARE* were indicated by the extended Langmuir compared to other models. While, the extended Langmuir–Freundlich model was characterized by a low %*ARE* for the biosorption of Fe(III) from a binary mixture, which indicated a better fitting. The maximum adsorption capacity (*q*_*m*_) for Fe(III) as indicated by the extended Langmuir and the extended Langmuir–Freundlich equations was higher than that of Fe(II) (Table 5).

**Table 5:** Isotherm parameters for the adsorption of Fe(II) and Fe(III) from binary system.

Isotherm	Parameters	F(II)	Fe(III)
Extended Langmuir	*q*_*m,i*_ (mg g^−1^)	1.96	3.54
	*K*_*LF,i*_ (L mg^−1^)	3.42	0.42
	*R*^2^	0.959	1.000
	*ARE* (%)	2.96	5.31
Extended Freundlich	*X*_*i*_	0.71	14.79
	*Y*_*i*_	0.00	0.00
	*Z*_*i*_	0.05	1.05
	*R*^2^	0.772	1.000
	*ARE* (%)	63.09	58.53
Extended Langmuir–Freundlich	*q*_*m*_*,*_*i*_ (mg g^−1^)	4.29	9.64
	*K*_*LF*_*,*_*i*_ (L mg^−1^)	5.92	5.95
	*n*_*i*_	1.27	0.020
	*R*^2^	0.987	0.990
	*ARE* (%)	18.39	1.94

### Distribution coefficient and selectivity of the CNC/GO film

The distribution coefficient (*K*_*d*_, L kg^−1^) was calculated using the following equation:

27 $${K}_{d}=\frac{{q}_{e}}{{C}_{e}}\times 1000$$

where *q*_*e*_ (mg g^−1^) and *C*_*e*_ (mg L^−1^) are the amount adsorbed and iron concentration at equilibrium, respectively. This parameter indicates the metal concentration in the solid phase to its concentration in the solution at equilibrium. The estimated *K*_*d*_ values at initial iron concentration of 15 mg L^−1^ in a single system was 251.3 and 309.4 L kg^−1^ for Fe(II) and Fe(III), respectively. In the binary system the distribution coefficient *K*_*d*_ for Fe(II) was estimated to be 3072.7, 10,971.4 and 2250.0 L kg^−1^ at Fe(II):Fe(III) ratios of 1:2, 1:1 and 2:1, respectively, while the *K*_*d*_ of Fe(III) was 2000.0, 4457.1 and 1530.4 L kg^−1^ at Fe(II):Fe(III) ratios of 1:2, 1:1 and 2:1, respectively.

In order to investigate the selectivity of the developed adsorbent to the bivalent and trivalent iron from binary solution, the separation factor *α* was calculated as follows:

28 $$\alpha =\frac{{K}_{d} Fe(II)}{{K}_{d} Fe(III)}$$

The *α* values were 1.5, 2.46 and 1.47 at Fe(II):Fe(III) ratios of 1:2, 1:1 and 2:1, respectively. The values of α higher than 1 indicated the presence of a degree of selectivity towards Fe(II).

## Discussion

The seaweed biomass of *Ulva lactuca* was used as a source of cellulose nanocrystals and utilized as a cost-effective and eco-friendly material for the development of nanocomposite film containing graphene oxide. The web-like morphology of the extracted cellulose from *U. lactuca* is similar to the crude cellulose extracted from *Codium sinuosa* and *Amphiroa rigida* but different from that obtained from *U. fasciata* and *U. linza*^37^. The developed nanocellulose material exhibited a spherical to polygonal particles. Generally, morphology, size and different physico-chemical properties of cellulose depend on different factors such as source, extraction and processing conditions, and pre- or post-treatments. Therefore, different nanocellulose forms with different morphological and physico-chemical characteristics have been prepared from algal biomass, such as nanofibrils and rod-shaped nanocrystals^38^. The spherical cellulose nanocrystals have been developed from plant-derived cellulose^39^.

The FT-IR analysis indicated that the CNC/GO film contained significant functional groups such as O–H, C=O, C–H and C–O–C groups. The significant shifting in the wavenumbers of these functional groups implied their fundamental role in the adsorption of iron ions from aqueous solutions. In general, several mechanisms for the adsorption of heavy metals have been proposed including ion exchange, complexation, coordination, and micro-precipitation^40^. Accordingly, during the adsorption of Fe(II) and Fe(III), these mechanisms can act simultaneously with variable degrees depending on several physico-chemical conditions such as pH, metal concentration, adsorbent dosage, and temperature. In general, the main mechanism of adsorption may be related to electrostatic interaction/complexation, since the oxygen-containing functional groups can offer electrons for Fe(II) and Fe(III) sequestration. Furthermore, the shift in the electron rich C=C absorption of graphene oxide after the adsorption process indicated the presence of Fe-π interactions^41^.

In general, contact time is a crucial parameter that can directly affect the adsorption process^42^. In other words, the surface of the adsorbent material is gradually occupied by metal ions until it has a constant value of the adsorption capacity and cannot remove more metal ions from the solution. The adsorption efficiency of the developed CNC/GO film for Fe(III) was higher than Fe(II) in a single system, while in the binary mixture the adsorption of Fe(II) was relatively higher.

Based on the results of the BBD, pH and initial metal concentration were identified as the most important factors affecting the adsorption process, while the adsorbent dosage exhibited a non-significant effect. This result was consistent with the observations of Langeroodi and coauthors, who indicated through BBD analysis that pH and adsorbent weight is the most crucial factors influencing Fe(III) adsorption using nanocomposite^43^. In general, pH plays a fundamental role in the dissociation of the functional groups present in the adsorbent surface. The developed CNC/GO film was characterized by a pH_pzc_ of 7.55, thus it has a positively charged surface at pH < pH_pzc_. The adsorption efficiency of Fe(II) and Fe(III) were increased by increasing the initial pH values of the solution, and the maximum adsorption was obtained at ∼ pH 5. Increasing the pH facilitates the adsorption of ^−^OH ions on the surface of CNC/GO film, making it negatively charged and consequently the adsorption of iron ions was increased^2^. However, the concentration of dissolved iron decreases with increasing the pH owing to the precipitation of iron hydroxides. In general, the hydrolysis of Fe(III) begins at acidic conditions (pH > 3), while the hydrolysis of Fe(II) begins at circumneutral conditions (pH > 6). Additionally, the hydrolysis of Fe(III) is independent and not influenced by Fe(II) in the binary solution^44^. Accordingly, the predominant species at pH 5–6 are Fe(II) and Fe(OH)_2_^+^ for Fe(II) and Fe(III) aqueous solutions, respectively^45^. The kinetic mechanisms for the adsorption of Fe(II) and Fe(III) in single and binary systems were best described by the PSO model. This result implied that chemical adsorption is one of the main mechanisms for iron adsorption by the CNC/GO film^46^. Similarly, Dai and coauthors^47^ reported that the PSO was the predominant mechanism during the adsorption process of Fe(II) and Fe(III) using thiourea cross-linked chitosan. However, the satisfactory fitting of the PFO model may indicate a degree of physical adsorption, and that the adsorption process may be controlled by mass transfer of iron ions onto the biosorbent surface^25^. The *K*_2_ values obtained from the PSO model exhibited similar values for Fe(II) and Fe(III) in a single system. In addition, the *K*_2_ values were generally increased in the binary solutions, which indicated a faster adsorption process. Conversely, the PSO rate constants (*h*) values for Fe(II) were higher than Fe(III) in both single and binary solutions. This observation may be related to the difference in molecular size, since Fe(III) is hydrolyzed in water to Fe(OH)_2_^+^, while Fe(II) remains as free ions at pH 5–6^45^.

The Elovich model assumes that the binding sites of the adsorbent are heterogenous with diverse binding energies, and it elucidates the chemisorption process^26^. The Elovich equation showed satisfactory fitting for the adsorption of Fe(II) and Fe(III) in single system, but failed to describe the adsorption of Fe(III) when Fe(II) ions were present. As the *β* values increases above unity, the adsorption process becomes reversible^5^. Accordingly, the adsorption of Fe(II) from single system was irreversible.

On the other hand, the intra-particle diffusion model indicated that the adsorption of Fe(II) and Fe(III) from single and binary solutions using CNC/GO film is not only controlled by the intra-particle diffusion, but the film diffusion also played a remarkable role. The diffusion rate constants *K*_*i*1_ for Fe(II) were higher in single solution than binary mixture, while the opposite trend was observed in case of Fe(III). This result implied that the coexistence of Fe(III) ions in solution affected the diffusion rate of Fe(II).

The equilibrium data for the adsorption of Fe(II) and Fe(III) in single system showed satisfactory fitting to the Langmuir, Freundlich, Langmuir–Freundlich, Temkin and Dubinin-Radushkevich isotherms. In the binary system all the models showed satisfactory fitting, but the extended Langmuir and the Extended Freundlich isotherms exhibited the lowest error values for Fe(II) and Fe(III), respectively. In general, Langmuir model is based on the assumption of homogenous adsorbent with a monolayer coverage of the adsorbate molecules, while the Freundlich isotherm refers to multilayer and heterogeneous adsorption on the adsorbent surface^48^. Additionally, the Temkin isotherm is related to the adsorbent-adsorbate interactions and indicates that the heat of adsorption decreases linearly with the coverage. The Temkin constant (*b*_*T*_) showed positive values, which implied an exothermic adsorption of Fe(II) and Fe(III) on the surface of CNC/GO film^5^. Furthermore, the *b*_*T*_ values lower than 20 kJ mol^−1^ are indicative of electrostatic interactions of physical adsorption^49^. Accordingly, the estimated *b*_*T*_ values for Fe(II) and Fe(III) at pH 5 and 7 were lower than 20 kJ mol^−1^, reflecting a predominant physical mechanism. The Temkin equilibrium binding constant (*A*_*T*_) was relatively higher in case of Fe(III) adsorption, which may be related to high binding energy compared to Fe(II).

On the other hand, the Dubinin–Radushkevich isotherm can elucidate the mechanism of heterogeneous surface adsorption, with Gaussian distribution of energy^50^. The mean free energy (*E* < 8) values obtained from the D-R isotherm and the 1*/n* parameter of the Freundlich model (1*/n* < 1) implied that the adsorption of Fe(II) and Fe(III) using CNC/GO film is predominately physisorption through electrostatic interactions^25^, and agreed with the Temkin model. Similarly, Aniagor and coworkers^5^ observed that the adsorption of Fe(II) was controlled by physical adsorption using functionalized microcrystalline cellulose.

## Conclusion

The developed cellulose nanocrystals/graphene oxide film prepared from *U. Lactuca*-derived cellulose was found to be suitable for the bioadsorption of Fe(II) and Fe (III) from aqueous solution. The optimum conditions defined for the bioremoval of Fe(II) were pH 5.13, adsorbent dosage 7.93 g L^−1^ and Fe(II) concentration 15.39 mg L^−1^, which increased the efficiency of Fe(II) removal to 65.60%, while the biosorption of Fe(III) was enhanced to 69.92% at pH 5.0, adsorbent dosage 2 g L^−1^, and Fe(III) concentration 15.0 mg L^−1^ in single system. The adsorption efficiency was further promoted in the binary mixture. The removal of Fe(II) at ratio of 1:1 of Fe(II):Fe(III) was 95.48%, while in a case of Fe(III) the removal was 79.17% at ratio of 1:2. Pseudo-second-order kinetics provided the best fitting for the experimental data of Fe(II) and Fe(III) in both single and binary systems. According to the results of the Freundlich, D-R and Temkin isotherms, the predominant mechanism of Fe(II) and Fe(III) adsorption is physisorption. The FT-IR analysis revealed that oxygen-containing functional groups were prominent in the biosorption process of iron on the surface of CNC/GO film through electrostatic and Fe-π interactions. The results of the present study showed that the developed nanocomposite film can be effectively utilized as a low-cost, and ecofriendly adsorbent for the removal of Fe(II) and Fe(III) from contaminated water.

## Data Availability

The datasets used and/or analyzed during the current study are available from the corresponding author on reasonable request.

## References

[1] Yao M, Wang Z, Liu Y, Yang G, Chen J (2019). Preparation of dialdehyde cellulose graftead graphene oxide composite and its adsorption behavior for heavy metals from aqueous solution. Carbohydr. Polym..

[2] Kocaoba S (2020). Adsorption of Fe(II) and Fe(III) from aqueous solution by using sepiolite: Speciation studies with MINEQL+ computer program. Sep. Sci. Technol..

[3] Haldar D, Duarah P, Purkait MK (2020). MOFs for the treatment of arsenic, fluoride and iron contaminated drinking water: A review. Chemosphere.

[4] Zhang Z, Xiao C, Adeyeye O, Yang W, Liang X (2020). Source and mobilization mechanism of iron, manganese and arsenic in groundwater of Shuangliao City, Northeast China. Water.

[5] Aniagor CO, Abdel-Halim ES, Hashem A (2021). Evaluation of the aqueous Fe (II) ion sorption capacity of functionalized microcrystalline cellulose. J. Environ. Chem. Eng..

[6] Carolin CF, Kumar PS, Saravanan A, Joshiba GJ, Naushad M (2017). Efficient techniques for the removal of toxic heavy metals from aquatic environment: A review. J. Environ. Chem. Eng..

[7] Ngah WSW, Hanafiah MAKM (2008). Removal of heavy metal ions from wastewater by chemically modified plant wastes as adsorbents: A review. Bioresour. Technol..

[8] Abral H (2020). Transparent and antimicrobial cellulose film from ginger nanofiber. Food Hydrocoll..

[9] Chen YW, Lee HV, Juan JC, Phang S-M (2016). Production of new cellulose nanomaterial from red algae marine biomass Gelidium elegans. Carbohydr. Polym..

[10] Wahlström N (2020). Cellulose from the green macroalgae *Ulva lactuca*: Isolation, characterization, optotracing, and production of cellulose nanofibrils. Cellulose.

[11] Zaki M (2021). Microbial treatment for nanocellulose extraction from marine algae and its applications as sustainable functional material. Bioresour. Technol. Rep..

[12] Gomaa M, Al-Badaani AA, Hifney AF, Adam MS (2021). Industrial optimization of alkaline and bleaching conditions for cellulose extraction from the marine seaweed *Ulva lactuca*. J. Appl. Phycol..

[13] Kang Y-G, Vu HC, Chang Y-Y, Chang Y-S (2020). Fe (III) adsorption on graphene oxide: A low-cost and simple modification method for persulfate activation. Chem. Eng. J..

[14] Peng W, Li H, Liu Y, Song S (2017). A review on heavy metal ions adsorption from water by graphene oxide and its composites. J. Mol. Liq..

[15] Li F, Jiang X, Zhao J, Zhang S (2015). Graphene oxide: A promising nanomaterial for energy and environmental applications. Nano Energy.

[16] Cao Y, Li X (2014). Adsorption of graphene for the removal of inorganic pollutants in water purification: A review. Adsorption.

[17] Pinto SC (2020). Bacterial cellulose/graphene oxide aerogels with enhanced dimensional and thermal stability. Carbohydr. Polym..

[18] Chen X, Zhou S, Zhang L, You T, Xu F (2016). Adsorption of heavy metals by graphene oxide/cellulose hydrogel prepared from NaOH/urea aqueous solution. Materials (Basel).

[19] Paulchamy B, Arthi G, Lignesh BD (2015). A simple approach to stepwise synthesis of graphene oxide nanomaterial. J. Nanomed. Nanotechnol..

[20] Schneider CA, Rasband WS, Eliceiri KW (2012). NIH Image to ImageJ: 25 years of image analysis. Nat. Methods.

[21] Lee CM (2015). Hydrogen-bonding network and OH stretch vibration of cellulose: Comparison of computational modeling with polarized IR and SFG spectra. J. Phys. Chem. B.

[22] Khan TA, Nazir M, Khan EA (2013). Adsorptive removal of rhodamine B from textile wastewater using water chestnut (*Trapa natans* L.) peel: Adsorption dynamics and kinetic studies. Toxicol. Environ. Chem..

[23] Fawzy MA, Gomaa M (2021). Optimization of citric acid treatment for the sequential extraction of fucoidan and alginate from *Sargassum latifolium* and their potential antioxidant and Fe(III) chelation properties. J. Appl. Phycol..

[24] Fawzy MA, Gomaa M (2021). Low-cost biosorption of Methylene Blue and Congo Red from single and binary systems using *Sargassum latifolium* biorefinery waste/wastepaper xerogel: An optimization and modeling study. J. Appl. Phycol..

[25] Fawzy MA, Gomaa M (2020). Use of algal biorefinery waste and waste office paper in the development of xerogels: A low cost and eco-friendly biosorbent for the effective removal of congo red and Fe (II) from aqueous solutions. J. Environ. Manage..

[26] Hifney AF, Zien-Elabdeen A, Adam MS, Gomaa M (2021). Biosorption of ketoprofen and diclofenac by living cells of the green microalgae *Chlorella* sp. Environ. Sci. Pollut. Res..

[27] Ghaee A, Shariaty-Niassar M, Barzin J, Zarghan A (2012). Adsorption of copper and nickel ions on macroporous chitosan membrane: Equilibrium study. Appl. Surf. Sci..

[28] Sun L, Yu H, Fugetsu B (2012). Graphene oxide adsorption enhanced by in situ reduction with sodium hydrosulfite to remove acridine orange from aqueous solution. J. Hazard. Mater..

[29] Pan N (2013). Removal of Th4+ ions from aqueous solutions by graphene oxide. J. Radioanal. Nucl. Chem..

[30] Liu Z, Li X, Xie W, Deng H (2017). Extraction, isolation and characterization of nanocrystalline cellulose from industrial kelp (*Laminaria japonica*) waste. Carbohydr. Polym..

[31] Bhutiya PL, Misra N, Rasheed MA, Hasan SZ (2018). Nested seaweed cellulose fiber deposited with cuprous oxide nanorods for antimicrobial activity. Int. J. Biol. Macromol..

[32] Qi Y, Yang M, Xu W, He S, Men Y (2017). Natural polysaccharides-modified graphene oxide for adsorption of organic dyes from aqueous solutions. J. Colloid Interface Sci..

[33] Tohamy H-AS, El-Sakhawy M, Kamel S (2021). Carboxymethyl cellulose-grafted graphene oxide/polyethylene glycol for efficient Ni (II) adsorption. J. Polym. Environ..

[34] Cobas M, Sanromán MA, Pazos M (2014). Box–Behnken methodology for Cr (VI) and leather dyes removal by an eco-friendly biosorbent: *F. vesiculosus*. Bioresour. Technol..

[35] Fawzy MA (2020). Biosorption of copper ions from aqueous solution by *Codium vermilara*: Optimization, kinetic, isotherm and thermodynamic studies. Adv. Powder Technol..

[36] Cardoso SL, Costa CSD, da Silva MGC, Vieira MGA (2020). Insight into zinc (II) biosorption on alginate extraction residue: Kinetics, isotherm and thermodynamics. J. Environ. Chem. Eng..

[37] Salem, D. M. S. A. & Ismail, M. M. Characterization of cellulose and cellulose nanofibers isolated from various seaweed species. *Egypt. J. Aquat. Res.* (2021).

[38] Samyn, P., Pappa, M., Lama, S. & Vandamme, D. Algae for Nanocellulose Production. in 293–343 (2021). 10.1007/978-3-030-81557-8_13.

[39] Trilokesh C, Uppuluri KB (2019). Isolation and characterization of cellulose nanocrystals from jackfruit peel. Sci. Rep..

[40] Valentín-Reyes J, García-Reyes RB, García-González A, Soto-Regalado E, Cerino-Córdova F (2019). Adsorption mechanisms of hexavalent chromium from aqueous solutions on modified activated carbons. J. Environ. Manage..

[41] Yin G (2020). Novel Fe–Mn binary oxide-biochar as an adsorbent for removing Cd(II) from aqueous solutions. Chem. Eng. J..

[42] Zhang, Y., Zhao, J., Jiang, Z., Shan, D. & Lu, Y. Biosorption of Fe (II) and Mn (II) ions from aqueous solution by rice husk ash. *Biomed. Res. Int.* **2014**, (2014). 10.1155/2014/973095 PMC4058686 24982918

[43] Samadani Langeroodi N, Farhadravesh Z, Dehno Khalaji A (2018). Optimization of adsorption parameters for Fe (III) ions removal from aqueous solutions by transition metal oxide nanocomposite. Green Chem. Lett. Rev..

[44] Ruby C, Géhin A, Abdelmoula M, Génin J-MR, Jolivet J-P (2003). Coprecipitation of Fe (II) and Fe (III) cations in sulphated aqueous medium and formation of hydroxysulphate green rust. Solid State Sci..

[45] de Mello Gabriel GV (2021). The environmental importance of iron speciation in soils: evaluation of classic methodologies. Environ. Monit. Assess..

[46] Ngah WSW, Ab Ghani S, Kamari A (2005). Adsorption behaviour of Fe (II) and Fe (III) ions in aqueous solution on chitosan and cross-linked chitosan beads. Bioresour. Technol..

[47] Dai J, Ren F, Tao C (2012). Adsorption behavior of Fe (II) and Fe (III) ions on thiourea cross-linked chitosan with Fe (III) as template. Molecules.

[48] Mansoor SJ, Abbasitabar F (2020). Adsorption behavior of Fe (II) and Fe (III) ions on polyaniline coated sawdust: Batch and fixed-bed studies. Acta Chim. Slov..

[49] Soltani R, Marjani A, Shirazian S (2019). Facile one-pot synthesis of thiol-functionalized mesoporous silica submicrospheres for Tl(I) adsorption: Isotherm, kinetic and thermodynamic studies. J. Hazard. Mater..

[50] Günay A, Arslankaya E, Tosun I (2007). Lead removal from aqueous solution by natural and pretreated clinoptilolite: Adsorption equilibrium and kinetics. J. Hazard. Mater..

